# Evolution of psychosocial burden and psychiatric symptoms in patients with psychiatric disorders during the Covid-19 pandemic

**DOI:** 10.1007/s00406-021-01268-6

**Published:** 2021-05-03

**Authors:** Michael Belz, Philipp Hessmann, Jonathan Vogelgsang, Ulrike Schmidt, Mirjana Ruhleder, Jörg Signerski-Krieger, Katrin Radenbach, Sarah Trost, Björn H. Schott, Jens Wiltfang, Claus Wolff-Menzler, Claudia Bartels

**Affiliations:** 1 Department of Psychiatry and Psychotherapy, University Medical Center Goettingen, von-Siebold-Str. 5, 37075 Goettingen, Germany; 2 McLean Hospital, Harvard Medical School, Translational Neuroscience Laboratory, Belmont, MA USA; 3 Department of Psychiatry and Psychotherapy, University Hospital Bonn, Bonn, Germany; 4 School for Mental Health and Neuroscience, Department of Psychiatry and Neuropsychology, Maastricht University Medical Centre, Maastricht, The Netherlands; 5 Geriatric Psychiatry, University Department of Geriatric Medicine FELIX PLATTER, Basel, Switzerland; 6 German Center for Neurodegenerative Diseases (DZNE), Goettingen, Germany; 7 Leibniz Institute for Neurobiology, Magdeburg, Germany; 8 Neurosciences and Signaling Group, Institute of Biomedicine (iBiMED), Department of Medical Sciences, University of Aveiro, Aveiro, Portugal

**Keywords:** Coronavirus, SARS-CoV-2, Mental health, Psychosocial stress, Adjustment disorder

## Abstract

The Covid-19 pandemic highly impacts mental health worldwide. Patients with psychiatric disorders are a vulnerable risk population for worsening of their condition and relapse of symptoms. This study investigates the pandemic-related course of psychosocial burden in patients with pre-existing mental disorders. With the newly developed Goettingen psychosocial Burden and Symptom Inventory (Goe-BSI) psychosocial burden has been traced *retrospectively* (1) before the pandemic (beginning of 2020), (2) at its beginning under maximum lockdown conditions (March 2020), and (3) for the *current state* after maximum lockdown conditions (April/May 2020). The Goe-BSI also integrates the Adjustment Disorder New Module (ADNM-20), assesses general psychiatric symptoms, and resilience. A total of 213 patients covering all major psychiatric disorders (ICD-10 F0-F9) were interviewed once in the time range from April, 24th until May 11th, 2020. Across all diagnoses patients exhibited a distinct pattern with an initial rise followed by a decline of psychosocial burden (*p* < 0.001, partial η^2^ = 0.09; Bonferroni-corrected pairwise comparisons between all three time-points: *p* < 0.05 to 0.001). Female gender and high ADNM-20 scores were identified as risk factors for higher levels and an unfavorable course of psychosocial burden over time. Most psychiatric symptoms remained unchanged. Trajectories of psychosocial burden vary in parallel to local lockdown restrictions and seem to reflect an adaptive stress response. For female patients with pre-existing mental disorders and patients with high-stress responses, timely and specific treatment should be scheduled. With the continuation of the pandemic, monitoring of long-term effects is of major importance, especially when long incubation times for the development of mental health issues are considered.

**Supplementary Information:**

The online version contains supplementary material available at 10.1007/s00406-021-01268-6.

## Introduction

Starting in late 2019, the Covid-19 pandemic spread worldwide causing a steep increase in mortality and severe somatic complications in millions. As a secondary consequence, the pandemic’s dynamics together with social restrictions constitute a mental health threat of considerable magnitude, most likely resulting in psychological sequelae of hitherto unknown extent.

Thus, an increased incidence of psychiatric disorders is expected that may peak later than somatic cases and bear the risk of longer disease duration requiring specific treatment [1]. An emerging body of evidence already demonstrates increased rates of first-onset mental health disorders in healthcare professionals [2–8], SARS-CoV-2 infected patients [9–11], and in the general population [4, 7, 12–16].

Patients with pre-existing psychiatric conditions might be particularly vulnerable [17] and thus react with worsening, or relapse of symptoms potentially due to a reduced level of functioning and poorer availability of coping strategies [1]. A reduced access to psychiatric/psychotherapeutic services following the pandemic and treatment-challenging mental health conditions [17] may also contribute to a poor outcome. Accordingly, patients with psychiatric disorders have been found to be more susceptible to contracting Covid-19 [17, 18], to have lower life expectancies, and poorer physical health outcomes in general [19].

Despite this, only a few studies have addressed patients with pre-existing mental health disorders so far. In a systematic review [20], only two of 43 included studies addressed patients with mental health issues and concluded a worsening of their condition related to the pandemic: first, a questionnaire survey revealed a general deterioration of mental health in 20.9% of assessed patients with psychiatric disorders in China, but psychiatric diagnoses were not reported [21]. Second, the other study specifically devoted to patients with eating disorders in Spain found an increase in symptomatology and additional anxiety symptoms [22]. A recent study targeting the general population in Italy suggested a higher risk for developing severe depression and anxiety symptoms in respondents of an online survey with a self-reported history of mental health problems [23]. Similarly, higher levels of anxiety, depression, stress and insomnia were found in 76 patients compared to healthy controls during strict lockdown measures in China, and high rates of Post-traumatic Stress Disorder (PTSD) symptoms [24]. Yet, this patient sample was restricted to unipolar depression (F32, F33) and a small range of anxiety disorders (F41, F41.8).

Few studies have also investigated specific psychiatric subgroups. A higher risk for developing symptoms of anxiety and PTSD was found in pregnant women with a self-reported history of anxiety or depression [25]. Evidence regarding the influence on addictive behavior is yet inconclusive [26, 27]. Clinical deterioration seems to be pronounced in Obsessive-Compulsive Disorder, including an increased prevalence of suicidal ideations [28]. For adult Attention Deficit Hyperactivity Disorder, high levels of emotional distress were reported, but with an unclear association to pandemic-related changes [29].

Irrespective of the population investigated, most studies focused on symptoms of anxiety, depression, PTSD, stress, insomnia, and self-harm/suicidality related to the pandemic [2–6, 8, 12–16, 23–25, 28–32]. We suppose that only a minority has been confronted with traumatic events as defined in DSM 5 or ICD-10, but rather experienced a critical and enduring stressful life period resulting in symptoms of adjustment disorder (rather than in PTSD). However, adjustment disorder as mental health outcome of the Covid-19 pandemic has only received little attention [29, 31, 33].

In sum, pandemic-related data for psychosocial burden of psychiatric patient populations is very limited, and most importantly, information on its evolution in pre-existing mental health conditions during the pandemic is scarce. The aim of this study was to trace back the stress response of patients with current psychiatric disorders, starting from their current state in April/May 2020 back to time-points before and at the beginning of the pandemic. We thereby aimed (1) to investigate how the continuing pandemic and different lockdown regulations impact the course of psychosocial burden in (2) a patient sample with a broad spectrum of major psychiatric disorders, and (3) additionally focused on the assessment of adjustment disorder symptoms, general psychiatric symptoms and resilience.

## Material and methods

### Study sample

To cover a preferably wide spectrum of psychiatric diseases in this study, participants were eligible if they were (1) ≥ 18 years old, (2) treated in the Department of Psychiatry and Psychotherapy at the University Medical Center Goettingen, Germany between 10/2019 and 03/2020, (3) not currently hospitalized, and if (4) they had a current diagnosis within the spectrum of “mental and behavioral disorders” (ICD-10: F00-F99). All diagnoses, primary and secondary, as well as comorbid somatic diagnoses, were determined by their treating clinicians (psychiatric residents, board-certified psychiatrists, psychologists, or licensed psychotherapists). Primary diagnosis was defined as treatment diagnosis, i.e. the primary cause of psychiatric/psychotherapeutic consultation and treatment. Exclusion criteria comprised the inability to give informed consent. From a total of 5223 patients with psychiatric disorders consulting our clinic at least once during 10/2019 and 03/2020, 1003 patients with dementia were excluded a priori due to their inability to give informed consent. Of the remaining 4220 patients, a convenience sample of 316 eligible patients was approached, finally resulting in *N* = 213 study participants providing informed consent and study data (participation rate: 67.4%).

Due to contact restrictions during the pandemic, oral/verbal informed consent was obtained from all patients prior to the study, was witnessed and formally recorded. Written informed consent was obtained later. Consent was both obtained for participation in the study, and publication of individual anonymized data in a journal.

### Study design

Participants were interviewed via telephone at one time-point in the acute phase of maximum social restrictions (“lockdown”) during the Covid-19 pandemic in Lower Saxony, Germany, from April, 24^th^ until May, 11^th^, 2020 (please see Supplementary Table S2). All interviews were performed by highly qualified and specialized clinicians, and—in 80.3% of cases—interviews were conducted by the patients’ clinician/therapist. All interviewers underwent a rater training prior to data collection. In sum, 28 therapists participated in data collection.

### Study measures: the Goettingen psychosocial burden and symptom inventory (Goe-BSI)

For the present study, we developed the Goettingen psychosocial Burden and Symptom Inventory (Goe-BSI) to be applied as a standardized and structured telephone interview. In total, the interview contains 77 items covering the following sections: (1) clinical and demographic data, and Covid-19 related information (e.g., being tested for Covid-19 virus, being sent to quarantine, allocation to a Covid-19 risk group, current symptoms suspicious of a SARS-CoV-2 infection), (2) course of psychosocial burden during the pandemic, (3) symptoms of an adjustment disorder due to the pandemic (assessed by the Adjustment Disorder New Module – 20 Item version [ADNM-20] [34]), (4) general psychiatric symptoms, and (5) resilience.

Patients’ estimations for psychosocial stress, psychiatric symptoms and quality of life on a 10-point Likert scale were combined to measure psychosocial burden. All items were inversely scaled with lower scores indicating higher psychosocial burden (0: *It could not be worse*; 10: *It could not be better*). Participants were asked to rate these three items for their *current* state (April/May 2020), and retrospectively for a time *before* the pandemic (i.e. January/February 2020) and for the *beginning* phase of the pandemic when maximum lockdown regulations were active in Germany (mid-March, 2020). These three ratings allowed to generate a retrospective *pseudo*-course of psychosocial burden during the evolution of the pandemic (primary outcome). Cronbach’s α yielded good to excellent internal consistencies for all three-item groups: (1) *before* (α = 0.84), (2) at the *beginning* of the pandemic (α = 0.86), and for the *current* state (α = 0.90). Please refer to Supplementary Information S1 for additional information on validity for the primary outcome.

The ADNM-20 is an established and validated instrument to measure psychological reactions to stressful life events. The pandemic was pre-defined as stressor (chronic life event), and 20 items were to be answered on a frequency scale from 1 (*never*) to 4 (*often*) in relation to this stressor (sum score ranges from 20 to 80 points, ≥ 48 points denote high risk for adjustment disorder) [35].

A total of 22 items were included to assess general psychiatric symptoms across all ICD-10 F-axes in relation to the pandemic (e.g., “I have become more vigilant than before the corona-crisis”) with answers ranging from 0 (*strongly disagree*) to 10 (*strongly agree*). The same scale was used for two final items focusing on resilience (e.g., “The pandemic also holds opportunities for me.”). In some cases, free-response sections allowed for explaining self-reports in more detail.

The interview with a duration of approximately 30 min per patient is available in a paper pencil and an online version (LimeSurvey). Personal data were pseudonymized.

### Statistical analyses

IBM SPSS Statistics 26 was used for data analysis. For descriptive representation, we computed means (*M*), standard deviations (SD) and Pearson correlations (*r*)^1^ for metric variables. To analyze the *course of* psychosocial burden during the pandemic (primary outcome), we used multiple general linear models (GLM) for repeated measures. All models accounted for one level of dependency in our dataset: Three measurements were added as within-subjects factor; psychosocial burden (1) *before* the pandemic, (2) *at the beginning* of the pandemic, (3) at the present moment (*current state*). Additionally, we added multiple between-subjects factors (e.g., ICD-10 F-axes) and gender as covariate (please see results for a detailed description of each GLM). Missing data can be derived from degrees of freedom for each model. For multiple comparisons, *p*-values were corrected within each model, using the Bonferroni method (initial significance: *p* < 0.05, two-tailed). Exploratory analyses of the ADNM-20 sum score additionally included UNIANOVA and t-tests (please see results for details).

## Results

### Basic characteristics of the study sample

A total of *N* = 213 patients at the Department of Psychiatry and Psychotherapy, University Medical Center Goettingen, were included and underwent telephone interview using the Goe-BSI. The total sample covered an age range from 18 to 95 years (*M* = 42.24, SD = 16.93). 44.1% of participating patients (*n* = 94) were male, 42.7% (*n* = 91) were female. 13.1% (*n* = 28) were of non-binary gender and/or were diagnosed with a gender identity disorder (ICD-10: F64. *). Among the most frequent of the 56 main/primary ICD-10 diagnoses were the following mental disorders (see Table 1 for details): (1) F64.0 (13.1%, *n* = 28), (2) F33.2 (8.5%, *n* = 18), (3) F84.5 (7.0%, *n* = 15), (4) F20.0 (6.6%, *n* = 14), and (5) F33.1 (6.6%, *n* = 14). Categorized by F-axes, the five most frequent were (1) affective disorders (F3, 36.6%, *n* = 78), (2) neurotic, stress-related and somatoform disorders (F4, 16.4%, *n* = 35), (3) disorders of adult personality and behavior (F6, 16.4%, *n* = 35), (4) schizophrenia, schizotypal and delusional disorders (F2, 14.6%, *n* = 31), and (5) disorders of psychological development” (F8, 9.4%, *n* = 20). Overall, *n* = 16 patients (7.5%) had been tested for Covid-19 (*n* = 1 positive), *n* = 11 (5.2%) had been quarantined. A total of *n* = 73 patients (34.3%) belonged to one of the risk groups for a severe course of a SARS-CoV-2 infection. Please also see Table 1 for details of psychotropic medication.

**Table 1:** Clinical characterization of the study sample

(A) Main F-diagnoses (ICD-10)		
F20.0 *Paranoid schizophrenia*		14 (6.6%)
F25.1 *Schizoaffective disorder, depressive type*		8 (3.8%)
F31.3 *Bipolar affective disorder, manic episode*		12 (5.6%)
F32.2 *Severe depressive episode*		10 (4.7%)
F33.1 *Recurrent depressive disorder, moderate episode*		14 (6.6%)
F33.2 *Recurrent depressive disorder, severe episode*		18 (8.5%)
F41.2 *Mixed anxiety and depressive disorder*		7 (3.3%)
F43.1 *Post-traumatic stress disorder*		7 (3.3%)
F64.0 *Transsexualism*		28 (13.1%)
F84.5 *Asperger’s syndrome*		15 (7.0%)
Others		66 (31.0%)

(B) F-axes (ICD-10)		
F0 *Organic, including symptomatic, mental disorders*		5 (2.3%)
F1 *Mental and behavioral disorders due to psychoactive substance use*		6 (2.8%)
F2 *Schizophrenia, schizotypal and delusional disorders*		31 (14.6%)
F3 *Affective disorders*		78 (36.6%)
F4 *Neurotic, stress-related and somatoform disorders*		35 (16.4%)
F5 *Behavioral syndromes with physiological disturbances and physical factors*		2 (0.9%)
F6 *Disorders of adult personality and behavior*		35 (16.4%)
F8 *Disorders of psychological development*		20 (9.4%)
F9 *Behavioral and emotional disorders with onset in childhood and adolescence*		1 (0.5%)

(C) Psychotropic medication		
Antidepressant	SSRI	62 (29.1%)
	SNRI	0 (0.0%)
	SSNRI	34 (16.0%)
	Tricyclic	9 (4.2%)
	Tetracyclic	24 (11.3%)
	Others^1^	17 (8.0%)
	Combination^2^	27 (12.7%)
	None	96 (45.1%)
Antipsychotic	Typical	2 (0.9%)
	Atypical	74 (34.7%)
	Combination	1 (0.5%)
	None	138 (64.8%)
Other	Mood stabilizer	31 (14.6%)
	Anti-dementia	1 (0.5%)
	Benzodiazepine	12 (5.6%)

Frequency (%). **(A)** F-diagnoses *n* ≤ 5 are summarized as “others”; **(B)** allocation of all F-diagnoses to the corresponding F-axes; **(C)** frequencies of psychotropic medication adds up to > 100% due to combination therapies. *SSRI* = selective serotonin reuptake inhibitors; *SNRI* = serotonin and norepinephrine reuptake inhibitors; *SSNRI* = selective serotonin and norepinephrine reuptake inhibitors; ^1^category “other antidepressants” (serotonin modulator, dual serotonergic antidepressants, MAO-inhibitor, atypical); ^2^combination of two or more antidepressants; *N* = 213 patients

### Course of psychosocial burden (primary outcome)

#### Total sample

**Fig. 1:**
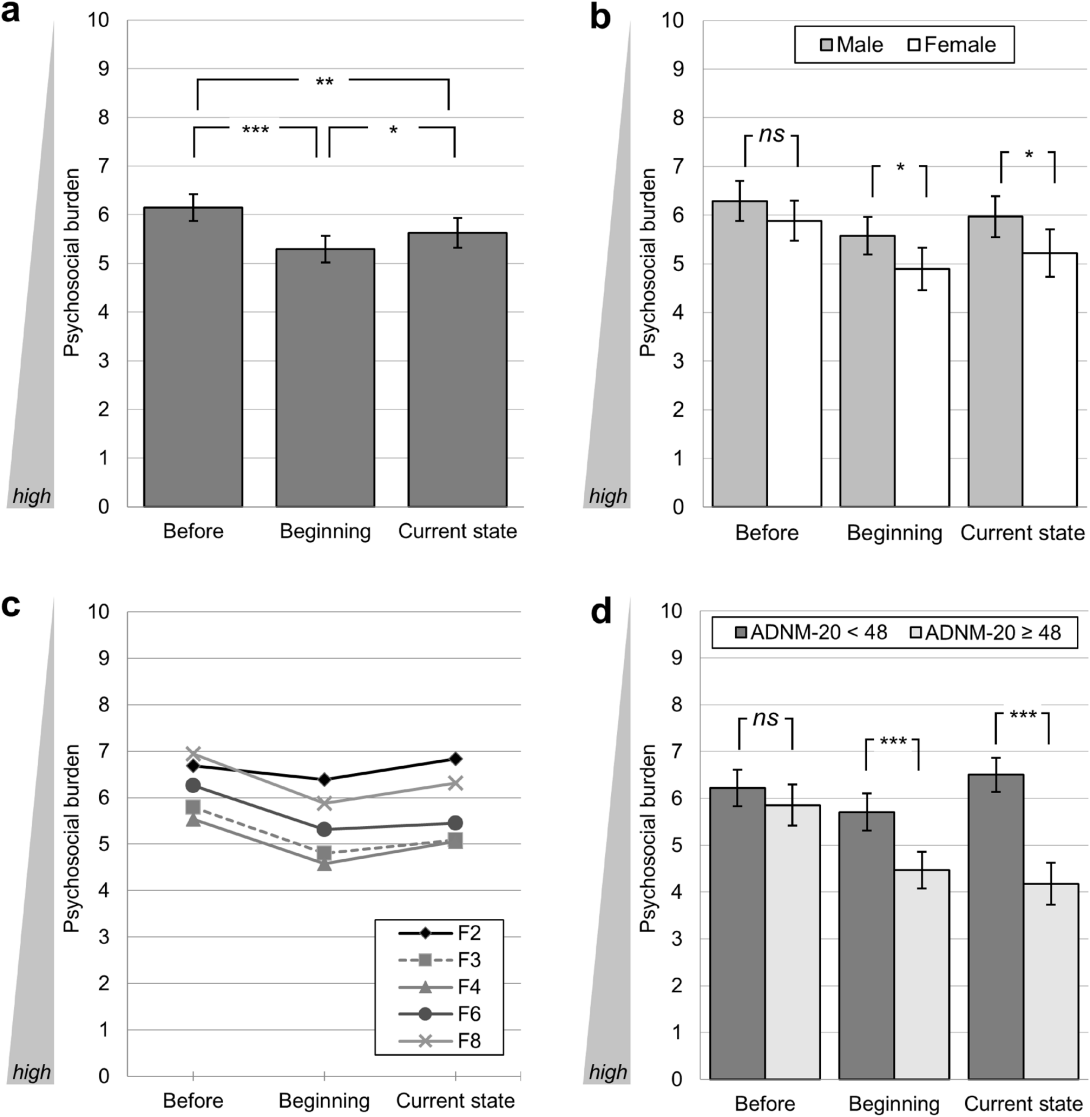
Course of psychosocial burden in patients with psychiatric disorders during different phases of the Covid-19 pandemic. **a** Course of the total sample (*N* = 210); differentiated by **b** gender (binary); **c** ICD-10 F-axes; **d** the ADNM-20 cut-off value indicating a high risk for adjustment disorder.* p < 0.05, ** *p* < 0.01, *** *p* < 0.001. Mean values with 95%-CIs (**a**, **b**, **d**) and Bonferroni corrected pairwise comparisons (**a**, **b**, **d**). Psychosocial burden is presented as mean of ratings on the 10-point Likert scales for psychosocial stress, psychiatric symptomatology, and quality of life. Ratings were performed retrospectively (*before* the pandemic: beginning of 2020; at the *beginning* of the pandemic: mid-March 2020), and for the *current* state (April/May 2020)

Psychosocial burden varied significantly between all time-points (GLM: *F*(2, 418) = 39.12, *p* < 0.001, partial η^2^ = 0.09, all Bonferroni-corrected pairwise comparisons *p* < 0.05 to 0.001; see Fig. 1a). Thereby, an increase of psychosocial burden from the time-point *before* the pandemic (*M* = 6.14, SD = 2.04) to the *beginning* of the pandemic/lockdown (*M* = 5.29, SD = 2.04) was followed by a relief of psychosocial burden over time (*current* state: *M* = 5.63, SD = 2.26). However, the psychosocial burden at the time of the interview (*current* state) was still increased compared to the time-point *before* the pandemic (please note the inverse scaling of psychosocial burden).

#### Gender differences

To analyze possible gender effects,^2^ an additional two-staged between-subjects factor (male vs. female) was added to the GLM (see Fig. 1b). In general, female patients showed significantly higher psychosocial burden scores (GLM: *F*(1, 180) = 5.67, *p* = 0.018, partial η^2^ = 0.03) with significant differences in pairwise comparisons for the phase at the *beginning* (*M*_*Diff*_ = 0.68, *p* < 0.05) and for the *current* state (*M*_*Diff*_ = 0.75, *p* = 0.024). However, the course of psychosocial burden followed the same pattern of increase and relief as described above (no significant interaction between scales and gender: GLM: *F*(2, 360) = 0.74, *ns*).

#### Differences between ICD-10 F-axes

Differences between the ICD-10 F-axes F2, F3, F4, F6, and F8 were analyzed by adding a five-staged between-subjects factor to the GLM.^3^ Significant differences between F-axes related to the general level of psychosocial burden (GLM: *F*(4, 191) = 5.64, *p* < 0.001, partial η^2^ = 0.11, see Fig. 1c). Patients with F3-axis and F4-axis diagnoses showed the highest levels of psychosocial burden, but significance was reached only for the comparison to patients with F2-axis diagnoses (*p* < 0.01). The course of psychosocial burden did not differ between the F-axes in terms of an interaction effect (GLM: *F*(8, 382) = 0.74, *ns*), and so, again, the course was found to be identical to that of the total sample.

#### Risk groups by ADNM-20

To identify specific risk groups exhibiting an unfavorable course of their mental health condition as the pandemic continues, we divided the sample into groups at high (*n* = 82) vs. low risk (*n* = 129) for adjustment disorder by the proposed cut-off of 47.5. In an additional GLM, we added a two-staged between-subjects factor, respectively, and traced the course of psychosocial burden. Gender (binary) was added as covariate, due to high correlation with ADNM-20 scores (*r* = 0.322, *p* < 0.01). Both ADNM-20 groups differed significantly in the experience of psychosocial burden (GLM: *F*(1, 177) = 22.49, *p* < 0.001, partial η^2^ = 0.11). Moreover, the analysis also revealed a significant interaction effect (GLM: *F*(2, 354) = 23.07, *p* < 0.001, partial η^2^ = 0.12, Fig. 1d): Whereas patients at low risk for adjustment disorder showed a slow decrease in psychosocial burden, patients at high risk for adjustment disorder experienced a continuous increase of psychosocial burden over the course of the pandemic.

### Descriptive results and exploratory analyses of secondary outcomes

#### ADNM-20 sum score

**Fig. 2:**
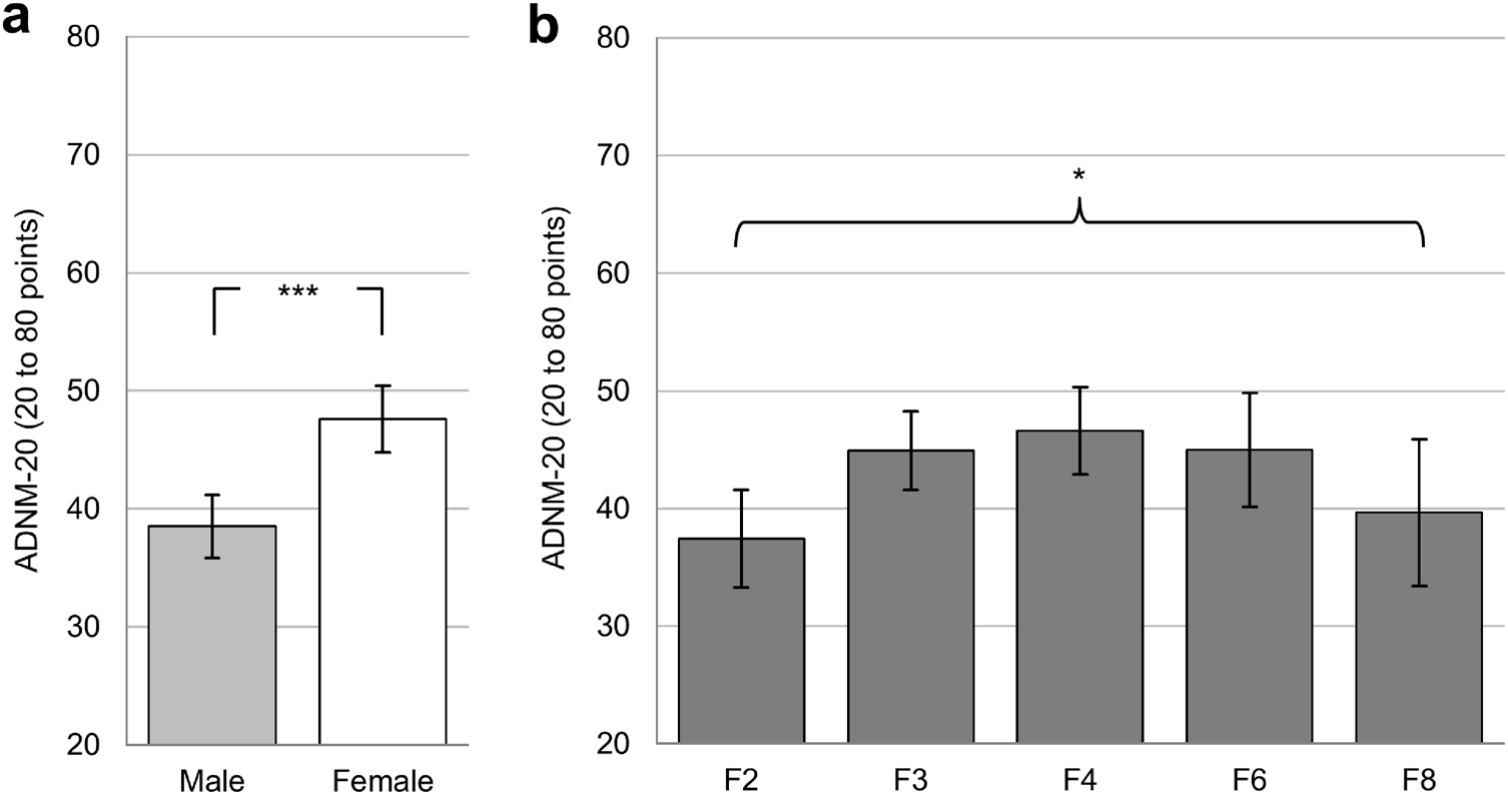
Pandemic-related symptom levels of adjustment disorder measured by the ADNM-20 in patients with psychiatric disorders. Differentiated by **a** gender (binary), *N* = 183; **b** by ICD-10 F-axes (*F2* to *F8*), *N* = 197. *p < 0.05, **p < 0.01, ***p < 0.001. Mean values with 95%-CIs and Bonferroni corrected pairwise comparisons for the *ADNM-20* sum score (range: 20 to 80 points)

For 211 of 213 patients complete data sets of the ADNM-20 were available (*M*_sum score_ = 42.84, SD = 14.07). As shown in Table 2, higher sum scores correlated with female gender (*r* = 0.322, *p* < 0.01), i.e. women showed a significantly higher ADNM-20 sum score (*M* = 47.61, SD = 13.78) compared to men (*M* = 38.51, SD = 14.17; *t*(181) = 4.57, *p* < 0.001; see Fig. 2a). UNINANOVA was used to analyze differences between the ICD-10 F-axes (see Fig. 2b) and revealed significant variation between the axes F2, F3, F4, F6, and F8 (*F*(4, 192) = 2.66, *p* = 0.034)^3^. However, none of those significances survived Bonferroni correction (*p* ≥ 0.072).

**Table 2:** Descriptive data for sociodemographic variables and secondary endpoints

*Variable*	1	2	3	4	5	6	7	8	9	10	11	*M* (SD)/Freq. (%)
*Sociodemographic variables*												
1. Age (in years)	–											42.24 (16.93)
2. Gender (male:female; %)	0.158^*^	–										94:91 (44.1%, 42.7%)
3. Living space (in m^2^)	0.092	0.004	–									92.00 (55.69)
4. Covid-19 risk group (yes:no; %)	− 0.515^**^	− 0.168^*^	0.041	–								73:140 (34.3%, 65.7%)
*ADNM-20*												
5. ADNM-20 sum score	− 0.040	0.322^**^	0.038	− 0.049	–							42.84 (14.07)
*Most pronounced psychiatric symptoms*												
6. Vigilance^a^	0.014	0.218^**^	0.048	− 0.118	0.459^**^	–						5.25 (3.27)
7. Media use^a^	− 0.147^*^	0.067	− 0.124	− 0.017	0.275^**^	0.174^*^	–					4.19 (3.58)
8. Observing disease symptoms of others^a^	− 0.122	0.128	0.082	0.089	0.431^**^	0.425^**^	0.274^**^	–				3.68 (3.39)
9. Poor drive^a^	− 0.056	0.300^**^	− 0.022	− 0.019	0.632^**^	0.191^**^	0.303^**^	0.215^**^	–			3.62 (3.11)
10. Self-observing of disease symptoms^a^	− 0.099	0.043	− 0.062	0.061	0.459^**^	0.429^**^	0.229^**^	0.608^**^	0.314^**^	–		3.53 (3.30)
*Resilience*												
11. Positive changes^b^	− 0.253^**^	− 0.050	0.124	0.259^**^	− 0.077	0.072	0.036	0.110	− 0.102	0.002	–	4.41 (3.59)
12. Opportunities^b^	− 0.198^**^	− 0.079	0.053	0.100	− 0.132	0.033	0.067	0.050	− 0.130	0.021	0.532^**^	3.14 (3.50)

Descriptive data presented as correlations, frequencies (Freq.), means (*M*), and standard deviations (SD). *p < 0.05. **p < 0.01. Captions: *Gender* (male = 1, female = 2); *risk group* for a severe course of Covid-19 (yes = 1, no = 2); *ADNM-20* sum score (20 to 80 points)

^a^most pronounced psychiatric symptoms: items rated from 0 to 10

^b^resilience: items rated from 0 to 10. (*N* = 170; *df* = 168 to *N* = 213; *df* = 211)

#### General psychiatric symptoms and resilience

15 of 22 pre-defined psychiatric symptoms did not, or only increased slightly (*M* ≤ 3) during the pandemic (please see Supplementary Table S3 for an English translation of all items). The five psychiatric symptoms with the strongest increase were (1) increased vigilance (*M* = 5.25), (2) more media use (*M* = 4.19), (3) paying attention to symptoms of others (*M* = 3.68), (4) poor drive (*M* = 3.62), and (5) paying attention to symptoms of oneself (*M* = 3.53). Besides positive correlations between these items (*r* = 0.174 to 0.608, *p* < 0.05 to < 0.01), older age was negatively correlated with media use (*r* = -0.147, *p* < 0.05), and female gender correlated with increased vigilance (*r* = 0.218, *p* < 0.01) and poor drive (*r* = 0.300, *p* < 0.01). Finally, the ADNM-20 sum score correlated positively with the top five items for changed psychiatric symptoms (*r* = 0.275 to 0.632, all *p* < 0.01). Please see Table 2 for details.

For both resilience items, responses scored *M* ≥ 3: (1) The perception that “for me, some things have changed in a positive way during the pandemic” and the perspective that “the pandemic also holds opportunities for me” were rated *M* = 4.41 and *M* = 3.14, respectively. Both items correlated positively with *r* = 0.532 (*p* < 0.01). Older age correlated negatively with both resilience items (*r* = -0.198 to -0.253, all *p* < 0.01). Not belonging to a Covid-19 risk group correlated positively with the perception of positive changes during the pandemic (*r* = 0.259, *p* < 0.01). Please see Table 2 for an overview.

## Discussion

In this study, we showed that psychosocial burden in patients with pre-existing and current mental health disorders followed a distinct pattern of increase and relief in the early phase of the Covid-19 pandemic and during maximum social restrictions in Germany. Particularly, female gender and a stress response with severe symptoms of adjustment disorder were identified as factors predicting higher psychosocial burden and an unfavorable course of psychosocial burden over time. Most psychiatric symptoms remained unchanged during the pandemic, except for higher awareness/vigilance to symptoms, media use, and poor drive. From our data, we have first preliminary evidence that most patients also exhibited resilience as indicated by ratings on positive changes and opportunities during the pandemic.

### Course of psychosocial burden

To our knowledge, this is the first study to report changes in psychosocial burden and psychiatric symptoms over time during the Covid-19 pandemic across a broad spectrum of psychiatric disorders. A recent longitudinal study presented a similar course with a reduction of stress-related symptoms within four weeks as the pandemic peaked, but focused on PTSD, anxiety, and depression in the general population [12]. Along the same line with an adaptation response to lockdown restrictions and shown by data from the general population, anxiety and/or depression levels declined during the first weeks of lockdown in UK [36, 37], even if initially increased in a very early phase of the pandemic [36]. Similar results from the Swiss Corona Stress Study also showed a decrease of anxiety levels (late April 2020 to May/June 2020) [38]. The COSMO study allows for comparisons with data from the German population before the outbreak of the pandemic. In contrast, this study found slightly increased psychological distress (anxiety and depression) but was confined to the first weeks of lockdown in Germany (late March to early April 2020, as published so far [7]). Another German study with population-based pre-pandemic reference data did not identify general differences in mental health conditions according to the WHO-5 well-being index and PHQ-D between 2018 and 2020 (late April to late May) [39]. Compared to these longitudinal data from the general population, patients with current psychiatric conditions in the present study showed higher baseline levels of symptoms before the pandemic but seemed to experience a stress response very similar to that of general population samples: The initial rise of psychosocial burden was followed by a decline, possibly reflecting adaption. In this matter, subsample analyses of a quota survey in UK isolated 27.2% of participants with self-reported, pre-existing psychiatric disorders showing initial higher levels of depression and anxiety, a lower well-being and a reduction of depressive symptoms from late March to early May 2020 [37]. However, longitudinal reference data from populations with current psychiatric disorders are hardly available so far.

At first glance, a course of psychosocial burden indicative of a normal stress response and only an increase in a minority of psychiatric symptoms in this particularly vulnerable population seems encouraging. One reason might be that patients show an adaptive stress response and employ resilience strategies to protect their individual well-being. However, despite generally elevated ADNM-20 sum scores in this population, one must consider that Germany showed moderate SARS-CoV-2 incidence rates compared to other countries. Even in the early months of the rising pandemic, the German healthcare system withstood the pandemic challenge in spring 2020 without collapsing. Also, only moderate restrictions were enacted in Germany compared to many other countries. Additionally, one might speculate that some patients even took advantage from their pre-existing low levels of functioning. Social withdrawal and isolation together with reduced leisure activities are common features of many psychiatric disorders, and such patients may have already been used to social deprivation. Furthermore, avoidance behavior and home confinement were legitimated by governmental regulations and may have contributed to the observed reduction in psychosocial burden. Treatment continuation, as realized via service hotlines, emergency contacts and early implementation of telepsychiatric services, might have also contributed to the rather small, partial and temporary impact on the psychosocial burden of the psychiatric population studied here.

### Risk groups

The identification of risk groups for clinical worsening is essential for timely and specific intervention. In these terms and with these first data, the Goe-BSI provides a reliable and valid measure (please see Supplementary Information S1) sensitive for change and particularly suitable for detection of risk groups for an unfavorable outcome. Being female has previously been identified as a risk factor for higher levels of anxiety, depression, and stress [7, 14, 23, 36–38]. Furthermore, our finding of a higher psychosocial burden in female patients was paralleled by elevated sum scores of the ADNM-20. Irrespective of gender, the ADNM-20 provided good discriminant validity and allowed to detect patients at risk for unfavorable trajectories of psychosocial burden. Importantly, 39% of the present patient sample were indicative of adjustment disorder compared to 44% of first-onset adjustment disorder in Covid-19 patients in China [40] and 16% in Iran [31].

As published so far, only two studies included assessments for adjustment disorder [14], while most projects focused on depression, anxiety, stress, and PTSD symptoms. Such, 8% of healthy medical staff members in Switzerland showed adjustment disorder according to the ADNM-20 [41]. Using the International Adjustment Disorder Questionnaire (IADQ), Rossi et al. found an increase in adjustment disorder symptoms in the general population during the first weeks of lockdown in Italy [14]. They also reported an association of quarantine and other recent Covid-19-related stressful life events with depression, anxiety, insomnia, stress, PTSD, and adjustment disorder symptoms. The lack of such relations in our sample might be due to lower rates of Covid-19 infections (*n* = 1) and the small proportion of patients with Covid-19-related stressors (e.g., quarantine, contact to infected individuals).

## Strengths and limitations

Data presented refers to outpatients only. Inpatients might have reacted with a milder stress response to pandemic-related changes as a result of different environmental conditions—specifically, hospitalization may have served as a protective factor. However, to keep the sample as homogenous as possible, this project focusses on outpatients who experienced environmental conditions more similar to those of the general population than an inpatient sample. Besides that, participants of this study continuously received treatment even though under different conditions (e.g. telemedicine). Trajectories of patients (1) without access to mental health services or (2) those who discontinued treatment might have resulted in a different and more pronounced stress response. Both aspects—hospitalization and absence of mental health care—may convey a potential inclusion bias.

Some ICD-10 diagnoses were underrepresented in this convenience sample, e.g. F0, F1, and F5. The low number of patients with organic mental disorders may be explained by the inability to give informed consent which is common in patients with dementia and related disorders. F5-diagnoses were most likely underrepresented because in Germany they are primarily treated by specialists for psychosomatic medicine rather than by psychiatrists. On the other hand, patients with gender identity disorders (F64.*) and adult Asperger’s syndrome (F84.5) were likely overrepresented in our sample, which results from the availability of specialized outpatient services in our department. Regarding substance use disorders (F1) a higher amount of relapses [27], and for psychotic disorders (F2) an increase of symptoms can be expected [42]. In this study, analyses have been performed in clusters of main/primary diagnosis. Hence, future analyses of types and amount of comorbid/secondary diagnoses may provide more exhaustive results. Still, our data showed that similar and robust patterns can be derived across genders and major ICD-10 F-axes.

Diagnostic accuracy is a major strength of our approach. Diagnoses do not rely on self-report like in many online surveys [23, 37], but were made by the patients’ treating clinicians. Furthermore, applying the Goe-BSI as telephone interview enabled us to obtain a nearly complete data set. In addition, with an approach combining current and retrospective data, we could create pseudo-trajectories of psychosocial burden during the pandemic. Certainly, retrospective estimation has to be interpreted cautiously and may be susceptible for confounding.

## Conclusion

Future prospective studies need to address whether a—presumably temporary—relief of psychosocial burden can be preserved or will finally revert to worsening of pre-existing mental health conditions as the pandemic continues. Detrimental long-term effects on mental health issues in patients with psychiatric disorders and the general population have to be expected considering long incubation times before relevant deterioration or newly developed mental health disorders can be observed. This scenario holds considerable challenges for the healthcare system and calls for sensitive tools to detect risk groups. Although a similar evolution pattern of psychosocial burden during an early phase of the pandemic was found across all F-axes in this study, patients with different psychiatric disorders would still require specialized treatment. Complementary, telemedicine already holds digital opportunities for patients with a particular risk, to prevent such worsening and might enable timely, intensified, and specific treatment as well as treatment continuity.

## Supplementary Information

Below is the link to the electronic supplementary material.

Supplementary file1 (DOCX 24 kb)

Supplementary file2 (DOCX 16 kb)

Supplementary file3 (DOCX 18 kb)

## Data Availability

The data that support the findings of this study are available on reasonable request from the corresponding author. The data are not publicly available due to privacy or ethical restrictions.

## Footnotes

1 To calculate a correlation between the binary variables *gender* (male vs. female) and *risk group* (yes vs. no), the mean square contingency coefficient (phi coefficient) was used and is reported in Supplementary Table S1 and Table 2 along with Pearson correlation coefficients for the remaining variables.

2 Explanation: Exclusion of patients with a Gender Identity Disorder (F64.0) due to confounding factors prohibiting valid assignment to biological/binary gender (e.g., hormone therapy, self-concept).

3 F-axes F0, F1, F5, and F9 had to be excluded from this analysis due to small sample size (*n* = 1 to 6).

## References

[1] Kesner L, Horáček J (2020). Three challenges that the COVID-19 pandemic represents for psychiatry. Br J Psychiatry.

[2] Liu S, Yang L, Zhang C (2020). Online mental health services in China during the COVID-19 outbreak. Lancet Psychiatry.

[3] Tan BYQ, Chew NWS, Lee GKH (2020). Psychological Impact of the COVID-19 pandemic on health care workers in Singapore. Ann Intern Med.

[4] Huang Y, Zhao N (2020). Generalized anxiety disorder, depressive symptoms and sleep quality during COVID-19 outbreak in China: a web-based cross-sectional survey. Psychiatry Res.

[5] Zhang H, Shi Y, Jing P (2020). Posttraumatic stress disorder symptoms in healthcare workers after the peak of the COVID-19 outbreak: a survey of a large tertiary care hospital in Wuhan. Psychiatry Res.

[6] Liang Y, Wu K, Zhou Y (2020). Mental health in frontline medical workers during the 2019 novel coronavirus disease epidemic in China: a comparison with the general population. Int J Environ Res Public Health.

[7] Gilan D, Röthke N, Blessin M (2020). Psychomorbidity, resilience, and exacerbating and protective factors during the SARS-CoV-2 pandemic. Dtsch Arztebl Int.

[8] Kramer V, Papazova I, Thoma A (2020). Subjective burden and perspectives of German healthcare workers during the COVID-19 pandemic. Eur Arch Psychiatry Clin Neurosci.

[9] Taquet M, Luciano S, Geddes JR, Harrison PJ (2020). Bidirectional associations between COVID-19 and psychiatric disorder: retrospective cohort studies of 62 354 COVID-19 cases in the USA. Lancet Psychiatry.

[10] Varatharaj A, Thomas N, Ellul MA (2020). Neurological and neuropsychiatric complications of COVID-19 in 153 patients: a UK-wide surveillance study. Lancet Psychiatry.

[11] Yamamoto V, Bolanos JF, Fiallos J (2020). COVID-19: review of a 21st Century pandemic from etiology to neuro-psychiatric implications. J Alzheimers Dis.

[12] Wang C, Pan R, Wan X (2020). A longitudinal study on the mental health of general population during the COVID-19 epidemic in China. Brain Behav Immun.

[13] Li J, Yang Z, Qiu H (2020). Anxiety and depression among general population in China at the peak of the COVID-19 epidemic. World Psychiatry.

[14] Rossi R, Socci V, Talevi D (2020). COVID-19 pandemic and lockdown measures impact on mental health among the general population in Italy. Front Psychiatry.

[15] Qiu J, Shen B, Zhao M (2020). A nationwide survey of psychological distress among Chinese people in the COVID-19 epidemic: implications and policy recommendations. Gen Psychiatr.

[16] McGinty EE, Presskreischer R, Han H, Barry CL (2020). Psychological distress and loneliness reported by US adults in 2018 and April 2020. JAMA.

[17] Li S, Zhang Y (2020). Mental healthcare for psychiatric inpatients during the COVID-19 epidemic. Gen Psychiatr.

[18] Yao H, Chen J-H, Xu Y-F (2020). Patients with mental health disorders in the COVID-19 epidemic. Lancet Psychiatry.

[19] Cullen W, Gulati G, Kelly BD (2020). Mental health in the COVID-19 pandemic. QJM.

[20] Vindegaard N, Benros ME (2020). COVID-19 pandemic and mental health consequences: systematic review of the current evidence. Brain Behav Immun.

[21] Zhou J, Liu L, Xue P (2020). Mental health response to the COVID-19 outbreak in China. Am J Psychiatry.

[22] Fernández-Aranda F, Casas M, Claes L (2020). COVID-19 and implications for eating disorders. Eur Eat Disord Rev.

[23] Fiorillo A, Sampogna G, Giallonardo V (2020). Effects of the lockdown on the mental health of the general population during the COVID-19 pandemic in Italy: results from the COMET collaborative network. Eur Psychiatry.

[24] Hao F, Tan W, Jiang L (2020). Do psychiatric patients experience more psychiatric symptoms during COVID-19 pandemic and lockdown? A case-control study with service and research implications for immunopsychiatry. Brain Behav Immun.

[25] Ravaldi C, Ricca V, Wilson A (2020). Previous psychopathology predicted severe COVID-19 concern, anxiety, and PTSD symptoms in pregnant women during “lockdown” in Italy. Arch Womens Ment Health.

[26] Martinotti G, Alessi MC, Di Natale C (2020). Psychopathological burden and quality of life in substance users during the COVID-19 lockdown period in Italy. Front Psychiatry.

[27] Dubey MJ, Ghosh R, Chatterjee S (2020). COVID-19 and addiction. Diabetes Metab Syndr.

[28] Benatti B, Albert U, Maina G (2020). What happened to patients with obsessive compulsive disorder during the COVID-19 pandemic? A multicentre report from tertiary clinics in Northern Italy. Front Psychiatry.

[29] Adamou M, Fullen T, Galab N (2020). Psychological effects of the COVID-19 imposed lockdown on adults with attention deficit/hyperactivity disorder: cross-sectional survey study. JMIR Form Res.

[30] Shi L, Lu Z-A, Que J-Y (2020). Prevalence of and risk factors associated with mental health symptoms among the general population in China during the coronavirus disease 2019 pandemic. JAMA Netw Open.

[31] Zarghami A, Farjam M, Fakhraei B (2020). A report of the telepsychiatric evaluation of SARS-CoV-2 patients. Telemed J E Health.

[32] Jefsen OH, Rohde C, Nørremark B, Østergaard SD (2020). COVID-19-related self-harm and suicidality among individuals with mental disorders. Acta Psychiatr Scand.

[33] Kazlauskas E, Quero S (2020). Adjustment and coronavirus: how to prepare for COVID-19 pandemic-related adjustment disorder worldwide?. Psychol Trauma.

[34] Einsle F, Köllner V, Dannemann S, Maercker A (2010). Development and validation of a self-report for the assessment of adjustment disorders. Psychol Health Med.

[35] Lorenz L, Bachem RC, Maercker A (2016). The adjustment disorder-new module 20 as a screening instrument: cluster analysis and cut-off values. Int J Occup Environ Med.

[36] Fancourt D, Steptoe A, Bu F (2020). Trajectories of anxiety and depressive symptoms during enforced isolation due to COVID-19 in England: a longitudinal observational study. Lancet Psychiatry.

[37] O’Connor RC, Wetherall K, Cleare S (2020). Mental health and well-being during the COVID-19 pandemic: longitudinal analyses of adults in the UK COVID-19 mental health & wellbeing study. Br J Psychiatry.

[38] de Quervain D, Aerni A, Amini E (2020). The swiss corona stress study. Open Sci Framework.

[39] Kuehner C, Schultz K, Gass P (2020). Psychisches Befinden in der Bevölkerung während der COVID-19-Pandemie. Psychiatr Prax.

[40] Xie Q, Fan F, Fan X-P (2020). COVID-19 patients managed in psychiatric inpatient settings due to first-episode mental disorders in Wuhan, China: clinical characteristics, treatments, outcomes, and our experiences. Transl Psychiatry.

[41] Krammer S, Augstburger R, Haeck M, Maercker A (2020). Adjustment disorder, depression, stress symptoms, corona related anxieties and coping strategies during the Corona Pandemic (COVID-19) in Swiss medical staff. Psychother Psychosom Med Psychol.

[42] Brown E, Gray R, Lo Monaco S (2020). The potential impact of COVID-19 on psychosis: a rapid review of contemporary epidemic and pandemic research. Schizophr Res.

