# Supplementary material for: Evolution of psychosocial burden and psychiatric symptoms in patients with psychiatric disorders during the Covid-19 pandemic
Source: Eur Arch Psychiatry Clin Neurosci. 2021 May 3;272(1):29–40. doi: 10.1007/s00406-021-01268-6 (PMC8092366; doi:10.1007/s00406-021-01268-6)
Supplement: Supplementary file 1 — Supplementary file1 (DOCX 24 kb) [file 406_2021_1268_MOESM1_ESM.docx]

**Supplementary Information S1**

**Title:** Evolution of psychosocial burden and psychiatric symptoms in patients with psychiatric disorders during the Covid-19 pandemic

**Journal:** European Archives of Psychiatry and Clinical Neuroscience

Michael Belz PhD^1^, Philipp Hessmann PhD^1^, Jonathan Vogelgsang PhD^1,2^, Ulrike Schmidt PhD^1,3,4^, Mirjana Ruhleder PhD^1^, Jörg Signerski-Krieger PhD^1^, Katrin Radenbach PhD^1^, Sarah Trost PhD^1,5^, Björn H. Schott PhD^1,6,7^, Prof. Jens Wiltfang^1,6,8^, Claus Wolff-Menzler PhD^1^, Claudia Bartels PhD^1^**^*^**

^1^Department of Psychiatry and Psychotherapy, University Medical Center Goettingen, Germany

^2^McLean Hospital, Harvard Medical School, Translational Neuroscience Laboratory, Belmont, MA, USA

^3^Department of Psychiatry and Psychotherapy, University Hospital Bonn, Germany

^4^Maastricht University Medical Centre, School for Mental Health and Neuroscience, Department of Psychiatry and Neuropsychology, Maastricht, The Netherlands

^5^Geriatric Psychiatry, University Department of Geriatric Medicine FELIX PLATTER, Basel, Switzerland

^6^German Center for Neurodegenerative Diseases (DZNE), Goettingen, Germany

^7^Leibniz Institute for Neurobiology, Magdeburg, Germany

^8^Neurosciences and Signaling Group, Institute of Biomedicine (iBiMED), Department of Medical Sciences, University of Aveiro, Aveiro, Portugal

***Corresponding author:** Claudia Bartels, Department of Psychiatry and Psychotherapy, University Medical Center Goettingen, von-Siebold-Str. 5, D-37075 Goettingen, Germany, [claudia.bartels@med.uni-goettingen.de](mailto:claudia.bartels@med.uni-goettingen.de), +49 551 3914397

**Supplementary Information S1**

**Reliability of the primary outcome scale “psychosocial burden” (Goe-BSI)^[[1]](#footnote-1)^**

Cronbach’s α yielded good to excellent internal consistencies for all three time-points of psychosocial burden: (1) *before* (α = 0.84), (2) at the *beginning* of the pandemic (α = 0.86), and for the *current state* (α = 0.90), indicative of a good reliability.

**Validity of the primary outcome scale “psychosocial burden” (Goe-BSI)^1^**

Please see Table S1 for correlations between the items for psychosocial burden and demographic variables, as well as the ADNM-20 sum score.

**Construct validity.** The three time-points describing the absence of burden (1) *before* the pandemic, (2) at the *beginning* of the pandemic, and (3) *current state* (0 to 10) were positively correlated (*r* = 0.438 to 0.705, *p* < 0.01). In line with the expectation, that the amount of psychosocial burden should be related depending on the temporal distance of the time-points, we found the strongest correlation between both retrospective measures (*before* vs. *beginning* of the pandemic, *r* = 0.705, *p* < 0.01), followed by the *current* *state* vs. the *beginning* of the pandemic (*r* = 0.577, *p* < 0.01). The lowest correlation was found for the two time-points with the highest temporal distance (*before* the pandemic vs. *current state*, *r* = 0.438, *p* < 0.01).

**Concurrent validity (ADNM-20).** The ADNM-20 sum score (range 20-80 with high scores denoting high symptom levels of an Adjustment Disorder) was coded inversely in comparison to the rating of psychosocial burden and thus, was negatively correlated with all three time-points of psychosocial burden. The highest correlation could be found for the time-point *current state* (*r* = -0.588, *p* < 0.01), as this current state was also measured with the ANDM-20. It was expectably lower as the temporal distance rose (ADNM-20 vs. *beginning* of the pandemic: *r* = -0.369, *p* < 0.01; ANDM-20 vs. *before* the pandemic: *r* = -0.117). In line with the ADNM-20 and other symptom-oriented measures, women scored higher for psychosocial burden than men (GLM: *F*(1, 180) = 5.67, *p* = 0.018, partial η^2^ = 0.03).

**Supplementary data for the primary outcome “psychosocial burden” and sociodemographic data**

Age and living space did not correlate significantly with the items for psychosocial burden. However, psychosocial burden was significantly correlated with being female at the *beginning* of the pandemic (*r* = -0.164, *p* < 0.05), and at the *current state* (*r* = -0.170, *p* < 0.05). Being in a Covid-19 risk group was significantly correlated with psychosocial burden at the *beginning* of the pandemic (*r* = -0.139, *p* < 0.05).

**Supplementary Table S1** Correlations with the course of psychosocial burden in patients with psychiatric disorders

| *Variable* | 1 | 2 | 3 | 4 | 5 | 6 | 7 | *M* (SD) / Freqencies (%) | | |
| --- | --- | --- | --- | --- | --- | --- | --- | --- | --- | --- |
| **Sociodemographic variables** | | | | | | | | | |  |
| 1. Age (in years) | – |  |  |  |  |  |  | 42.24 (16.93) | | |
| 2. Gender (male:female; %) | 0.158^*^ | – |  |  |  |  |  | 94:91 (44.1%, 42.7%) | | |
| 3. Living space (in m²) | 0.092 | 0.004 | – |  |  |  |  | 92.00 (55.69) | | |
| 4. Covid-19 risk group (yes:no; %) | -0.515^**^ | -0.168^*^ | 0.041 | – |  |  |  | 73:140 (34.3%, 65.7%) | | |
| **ADNM-20** | | | | | | | | |  |  |
| 5. ADNM-20 sum score | -0.040 | 0.322^**^ | 0.038 | -0.049 | – |  |  | 42.84 (14.07) | | |
| **Psychosocial burden** | | | | | | | | |  |  |
| 6^1^. Before the pandemic | 0.019 | -0.098 | 0.063 | 0.099 | -0.117 | – |  | 6.15 (2.03) | | |
| 7^1^. Beginning of the pandemic/lockdown | 0.005 | -0.164^*^ | -0.013 | 0.139^*^ | -0.369^**^ | 0.705^**^ | – | 5.30 (2.03) | | |
| 8^1^. Current state | 0.025 | -0.170^*^ | 0.018 | 0.071 | -0.588^**^ | 0.438^**^ | 0.577^**^ | 5.62 (2.25) | | |

*Notes.* Data presented as correlations, frequencies, means (*M*), and standard deviations (SD). ** p* < 0.05. ** *p* < 0.01. Captions: *Gender* (male = 1, female = 2); *risk group* for a severe course of Covid-19 (yes = 1, no = 2); *ADNM-20* sum score (20 to 80 points); ^1^psychosocial burden: items rated from 0 to 10, low scores denote high psychosocial burden*.* (*N* = 170; *df* = 168 to *N* = 213; *df* = 211).

1. Goettingen psychosocial Burden and Symptom Inventory [↑](#footnote-ref-1)
