# Supplementary material for: Evolution of psychosocial burden and psychiatric symptoms in patients with psychiatric disorders during the Covid-19 pandemic
Source: Eur Arch Psychiatry Clin Neurosci. 2021 May 3;272(1):29–40. doi: 10.1007/s00406-021-01268-6 (PMC8092366; doi:10.1007/s00406-021-01268-6)
Supplement: Supplementary file 2 — Supplementary file2 (DOCX 16 kb) [file 406_2021_1268_MOESM2_ESM.docx]

**Supplementary Table S2**

**Title:** Evolution of psychosocial burden and psychiatric symptoms in patients with psychiatric disorders during the Covid-19 pandemic

**Journal:** European Archives of Psychiatry and Clinical Neuroscience

Michael Belz PhD^1^, Philipp Hessmann PhD^1^, Jonathan Vogelgsang PhD^1,2^, Ulrike Schmidt PhD^1,3,4^, Mirjana Ruhleder PhD^1^, Jörg Signerski-Krieger PhD^1^, Katrin Radenbach PhD^1^, Sarah Trost PhD^1,5^, Björn H. Schott PhD^1,6,7^, Prof. Jens Wiltfang^1,6,8^, Claus Wolff-Menzler PhD^1^, Claudia Bartels PhD^1^**^*^**

^1^Department of Psychiatry and Psychotherapy, University Medical Center Goettingen, Germany

^2^McLean Hospital, Harvard Medical School, Translational Neuroscience Laboratory, Belmont, MA, USA

^3^Department of Psychiatry and Psychotherapy, University Hospital Bonn, Germany

^4^Maastricht University Medical Centre, School for Mental Health and Neuroscience, Department of Psychiatry and Neuropsychology, Maastricht, The Netherlands

^5^Geriatric Psychiatry, University Department of Geriatric Medicine FELIX PLATTER, Basel, Switzerland

^6^German Center for Neurodegenerative Diseases (DZNE), Goettingen, Germany

^7^Leibniz Institute for Neurobiology, Magdeburg, Germany

^8^Neurosciences and Signaling Group, Institute of Biomedicine (iBiMED), Department of Medical Sciences, University of Aveiro, Aveiro, Portugal

***Corresponding author:** Claudia Bartels, Department of Psychiatry and Psychotherapy, University Medical Center Goettingen, von-Siebold-Str. 5, D-37075 Goettingen, Germany, [claudia.bartels@med.uni-goettingen.de](mailto:claudia.bartels@med.uni-goettingen.de), +49 551 3914397

**Supplementary Table S2** Lockdown restrictions in Lower Saxony, Germany, March and May 2020

| **(A)** Middle/late March 2020^1^ | **(B)** Starting from May 11, 2020^2^ |
| --- | --- |
| **Public sector** | |
| 1.5m distance | |
| Permission to go out for everyday commute, purchases for everyday needs, doctor appointments, individual outdoor sports and similar acts | |
| Museums, theaters etc. closed | |
| Schools and children day-care centers closed | Stepwise re-opening of schools (hybrid lessons) and children day-care centers |
| Shops closed except for supermarkets, drug stores, filling stations | Re-opening of shops under strict hygienic restrictions |
| Restaurants, hotels, service providers (e.g., hairdresser) closed | Stepwise re-opening of restaurants under strict hygienic restrictions, hotels, service providers (e.g., hairdresser) |
| Sports complexes closed, sports activities prohibited (except for individual outdoor sports) | Re-opening of popular/leisure sports offers (outdoor only) |
| **Individual contacts** | |
| Limited to own household + 1 person of a different household | Limited to own household + multiple persons of a different household |
| **Psychiatry, UMG**^3^ | |
| Inpatient treatment reduced | Stepwise return to regular inpatient treatment |
| Day-care clinics closed | Slow re-opening of day-care clinics |
| No outpatient contacts, hotlines,  establishment of telemedicine services | Sporadic outpatient contacts + telemedicine contacts |
| Emergency contacts only | Emergency contacts as usual |
| No visitors | One visitor per patient |

*Notes.* Contact between persons besides the same household had to be reduced to a “minimal” amount in general. This was not specified due different regulations in the federal states of Germany and multiple changes in this period.

^1^<https://www.niedersachsen.de/download/153376/Allgemeinverfuegung_des_Niedersaechsischen_Gesundheitsministeriums_zur_Beschraenkung_von_Sozialen_Kontakten_vom_22.03.2020.pdf>

^2^<https://www.ms.niedersachsen.de/startseite/aktuelles/presseinformationen/niedersachsische-verordnung-zur-bekampfung-der-corona-pandemie-tritt-am-montag-in-kraft-neue-phase-mit-weiteren-lockerungen-startet-188195.html>

^3^Department of Psychiatry and Psychotherapy, University Medical Center Goettingen, Germany
