# Supplementary material for: Evolution of psychosocial burden and psychiatric symptoms in patients with psychiatric disorders during the Covid-19 pandemic
Source: Eur Arch Psychiatry Clin Neurosci. 2021 May 3;272(1):29–40. doi: 10.1007/s00406-021-01268-6 (PMC8092366; doi:10.1007/s00406-021-01268-6)
Supplement: Supplementary file 3 — Supplementary file3 (DOCX 18 kb) [file 406_2021_1268_MOESM3_ESM.docx]

**Supplementary Table S3**

**Title:** Evolution of psychosocial burden and psychiatric symptoms in patients with psychiatric disorders during the Covid-19 pandemic

**Journal:** European Archives of Psychiatry and Clinical Neuroscience

Michael Belz PhD^1^, Philipp Hessmann PhD^1^, Jonathan Vogelgsang PhD^1,2^, Ulrike Schmidt PhD^1,3,4^, Mirjana Ruhleder PhD^1^, Jörg Signerski-Krieger PhD^1^, Katrin Radenbach PhD^1^, Sarah Trost PhD^1,5^, Björn H. Schott PhD^1,6,7^, Prof. Jens Wiltfang^1,6,8^, Claus Wolff-Menzler PhD^1^, Claudia Bartels PhD^1^**^*^**

^1^Department of Psychiatry and Psychotherapy, University Medical Center Goettingen, Germany

^2^McLean Hospital, Harvard Medical School, Translational Neuroscience Laboratory, Belmont, MA, USA

^3^Department of Psychiatry and Psychotherapy, University Hospital Bonn, Germany

^4^Maastricht University Medical Centre, School for Mental Health and Neuroscience, Department of Psychiatry and Neuropsychology, Maastricht, The Netherlands

^5^Geriatric Psychiatry, University Department of Geriatric Medicine FELIX PLATTER, Basel, Switzerland

^6^German Center for Neurodegenerative Diseases (DZNE), Goettingen, Germany

^7^Leibniz Institute for Neurobiology, Magdeburg, Germany

^8^Neurosciences and Signaling Group, Institute of Biomedicine (iBiMED), Department of Medical Sciences, University of Aveiro, Aveiro, Portugal

***Corresponding author:** Claudia Bartels, Department of Psychiatry and Psychotherapy, University Medical Center Goettingen, von-Siebold-Str. 5, D-37075 Goettingen, Germany, [claudia.bartels@med.uni-goettingen.de](mailto:claudia.bartels@med.uni-goettingen.de), +49 551 3914397

**Supplementary Table S3** Item formulations (translated to English) and descriptive data: General psychiatric symptoms and resilience

| *Goe-BSI items* | *M* (SD) |
| --- | --- |
| **(A) General psychiatric symptoms** | |
| 1. “I have become more vigilant than before the corona-crisis.” | 5.25 (3.27) |
| 2. “Since the beginning of the crisis, I have spent more time on the internet or with media than before (except for home office).” | 4.19 (3.58) |
| 3. “I have been paying more attention to possible symptoms of illness in others since the crisis began.” | 3.68 (3.38) |
| 4. “Due to the crisis, I have less drive to undertake and tackle things.” | 3.62 (3.11) |
| 5. “I have been paying more attention to possible symptoms of illness in myself since the crisis began.” | 3.53 (3.30) |
| 6. “Since the beginning of the corona-crisis, I have been less physically active.” | 3.40 (3.55) |
| 7. “I don’t enjoy things the way I used to since the beginning of the crisis.” | 3.00 (3.00) |
| 8. “Feelings of anxiety have increased since the crisis began.” | 2.93 (3.16) |
| 9. “Since the beginning of the crisis, I eat more or less than before.” | 2.69 (3.31) |
| 10. “Since the beginning of the crisis, I have increasingly withdrawn emotionally from others. (not meant: social distancing).” | 2.62 (3.02) |
| 11. “I feel more anger or I am more aggressive since the crisis began.” | 2.17 (2.69) |
| 12. “My cognitive functions (orientation, comprehension, concentration, memory) have declined during the corona-crisis.” | 1.73 (2.81) |
| 13. “Since the beginning of the corona-crisis, I have had more physical symptoms than before.” | 1.66 (2.71) |
| 14. “Compared to the time before the crisis, I have more conflicts with other people.” | 1.43 (2.32) |
| 15. “Since the beginning of the crisis, I have had greater craving for addictive substances (alcohol, illicit drugs) than before.” | 0.64 (1.84) |
| 16. “Since the beginning of the crisis, I have been taking more pills.” | 0.62 (2.04) |
| 17. “Since the beginning of the crisis, I have felt watched and persecuted more often.” | 0.59 (1.63) |
| 18. “Since the beginning of the crisis, I consume more alcohol or illicit drugs.” | 0.51 (1.62) |
| 19. “Compared to the time before the crisis, physical or psychological violence in my partnership or family has increased.” | 0.44 (1.58) |
| 20. “Since the beginning of the crisis, I have felt more strongly that others are conspiring against me.” | 0.36 (1.19) |
| 21. “Since the beginning of the crisis, I have more often special perceptions that others do not have (e.g., hearing voices, seeing people or things).” | 0.30 (1.26) |
| 22. “Since the beginning of the crisis, I have been engaging in self-injurious behavior more frequently.” | 0.24 (1.14) |
| **(B) Resilience** | |
| 1. “For me, some things have changed in a positive way during the pandemic.” | 4.41 (3.59) |
| 2. “The pandemic also holds opportunities for me.” | 3.14 (3.50) |

*Notes.* English translation of Goe-BSI (Goettingen psychosocial Burden and Symptom Inventory) items for **(A)** general psychiatric symptoms and **(B)** resilience with means (*M*), and standard deviations (SD). All items were answered on a Likert scale from 0 to 10 (0 = “does not apply at all” to 10 = “fully applies”). The mean values within the categories **(A)** and **(B)** are sorted by size in descending order (*N* = 212 to *N* = 213).
